# The Spread of Venereal Disease

**Published:** 1920-10-02

**Authors:** 


THE PUBLIC WELL-BEING—*(continued)*.

### The Spread of Venereal Disease.

The first annual report of the Ministry of Health is an interesting document, especially in its revelation of the enormously increasing costs of the various medical services.

In child welfare, a branch of work in which considerable success has been achieved, local authorities and voluntary agencies received between them about half a million of money, compared with less than a quarter of a million in the previous year. But in this case of venereal disease, which is increasing instead of diminishing, the Ministry has spent nearly a quarter of a million on its " cure," while in the work of prevention the National Council has received substantial grants for propaganda purposes, the effect of which does not seem to have been particularly notable. During the year, up to March 31, 1919, grants amounting to £13,000 were made by the Local Government Board, and this year a grant not exceeding £20,000 was promised for " press advertisements, propaganda by cinema films, slides and exhibits, by pamphlets, posters and other literature." The sum actually paid was £15,000.

Upwards of 98,000 cases of venereal disease were reported for the first time in 1919. Of this number 15,500 were found not to be suffering from the disease, so that the number of new cases is 82,500. But that is not all. Attendances at the centres during 1919 numbered 1,003,000, as compared with 488,000 in 1918. It is fairly obvious that the increase is partly due to the growing tendency to seek treatment and to the more generally-known facilities offered, but it is obvious also from these figures that the means of prevention now employed are by no means near to achieving the end in view.

Very large sums, too, are being expended in the treatment of tuberculosis, though equally in this disease we seem to be still a very long way from obtaining a mastery over its incidence. Up to March 31 of the present year, a sum of £445,433 had been paid, and a sum of £893,999 promised, in aid of institutions in England for the treatment of the disease. There was, it is to be noted, a fall in tubercle last year. The number of notifications of the pulmonary form was 67,123, as compared with 79,025 in the previous year, and the number in respect of other " forms " was 17,775, against 20,215 in the previous year. The number of deaths (pulmonary) was 36,662, as against 46,077; and 9,650 (other forms), against 11,996. These figures as far as they go, are satisfactory.

